# Adipose tissue macrophages induce PPARγ-high FOXP3^+^ regulatory T cells

**DOI:** 10.1038/srep16801

**Published:** 2015-11-19

**Authors:** Toshiharu Onodera, Atsunori Fukuhara, Myoung Ho Jang, Jihoon Shin, Keita Aoi, Junichi Kikuta, Michio Otsuki, Masaru Ishii, Iichiro Shimomura

**Affiliations:** 1 Department of Metabolic Medicine, Osaka University Graduate School of Medicine, 2-2, Yamamdaoka, Suita, Osaka, Japan; 2 Academy of Immunology and Microbiology (AIM), Institute for Basic Science (IBS), Pohang 790-784, Korea; 3 Department of Immunology and Cell Biology, Osaka University Graduate School of Medicine & Frontier Biosciences, WPI-Immunology Frontier Research Center, 2-2, Yamamdaoka, Suita, Osaka, Japan; 4 JST, CREST, 5 Sanban-cho, Chiyoda-ku, Tokyo, Japan

## Abstract

Numerous regulatory T cells (Tregs) are present in adipose tissues compared with other lymphoid or non-lymphoid tissues. Adipose Tregs regulate inflammatory state and insulin sensitivity. However, the mechanism that maintains Tregs in adipose tissue remains unclear. Here, we revealed the contribution of adipose tissue macrophages (ATMs) to the induction and proliferation of adipose Tregs. ATMs isolated from mice under steady state conditions induced Tregs with high expression of PPARγ compared with splenic dendritic cells *in vitro*. Furthermore, ATMs from obese mice prompted the differentiation of PPARγ low Tregs. Adoptive transfer of ATMs induced differentiation and proliferation of Tregs, whereas depletion of ATMs by clodronate-liposome resulted in reduction of adipose Tregs, *in vivo*. Deficiency of anti-inflammatory adipocytokine, Adipoq, resulted in small proportions of ATMs and adipose Tregs without alteration of other immune cells *in vivo*. Therefore, these data suggest that the abundance of Tregs in adipose tissue could be partly attributed to the ability of ATMs to induce PPARγ-expressing Tregs.

Clinical and experimental studies have demonstrated that fat accumulation plays a key role in the development of various metabolic disorders^1^. An inflammatory state of white adipose tissue (WAT) has been highlighted in adipose tissues of obese human and obesity animal models, evident by extensive infiltration of immune cells, such as macrophages^2^, dendritic cells (DCs)^3^ and lymphocytes^4^. Accumulation of immunocytes in adipose tissues can potentially lead to a considerable amplification of the inflammatory process.

Regulatory T cells (Tregs) provide critical defense against inappropriate immune responses, such as allergy, inflammation, infection and tumorigenesis^5^,^6^. Typically, Tregs can suppress the activation and proliferation of effector T cells, and also influence the activities of the innate immune system^7^. Tregs are characterized by expression of forkhead box P3 (FOXP3). Classically, Tregs are divided into two populations; thymus-derived naturally occurring Tregs (tTregs) and peripherally-derived Tregs (pTregs). The former population is important in the control of immune activation toward self-antigens, which enables the regulation of a wide range of immune-mediated pathologies. On the other hand, pTregs can interact with foreign antigens as well as self-antigens and are required in peripheral tissues that are exposed continuously to exogenous antigens^8^.

Large numbers of Tregs are present in WAT of mice, accounting for almost 40% of T cells^9^. Their population in epididymal fat is markedly reduced in obese animals, and this reduction is closely associated with insulin resistance^10^. Peroxisome proliferator-activated receptor (PPAR) γ is a crucial molecular orchestrator of adipose Tregs accumulation and maintenance of their phenotype and function^10^. However, little is known about the mechanisms responsible for the presence of high proportion of Tregs in WAT.

Adiponectin is an adipocytokine secreted by adipocytes and has anti-diabetic and anti-inflammatory properties^11^. The adipose tissue stromal vascular fraction (SVF) is a rich source of preadipocytes, mesenchymal stem cells, endothelial progenitor cell, T cells, B cells, mast cells as well as adipose tissue macrophages (ATMs). The parabiosis models of wild type (WT) and Adipoq (−/−) demonstrated that adiponectin goes back to adipose tissue especially into adipose SVF^12^, suggesting that adiponectin exerts some effect on adipose SVF that contains immune cells such as macrophages and T cells. In this regard, however, the direct effect of adiponectin on adipose immune cells remains to be investigated.

The aim of the present study was to determine the mechanism that maintains Tregs in adipose tissue. Towards this goal, we examined the ability of ATMs to induce Tregs. The results showed that ATMs induce PPARγ-expressing Tregs and thus explain, at least in part, the abundance of Tregs in adipose tissue.

## Results

### Local proliferation of adipose tissue Tregs and phenotype of adipose tissue antigen presenting cells

**Figure 1:**
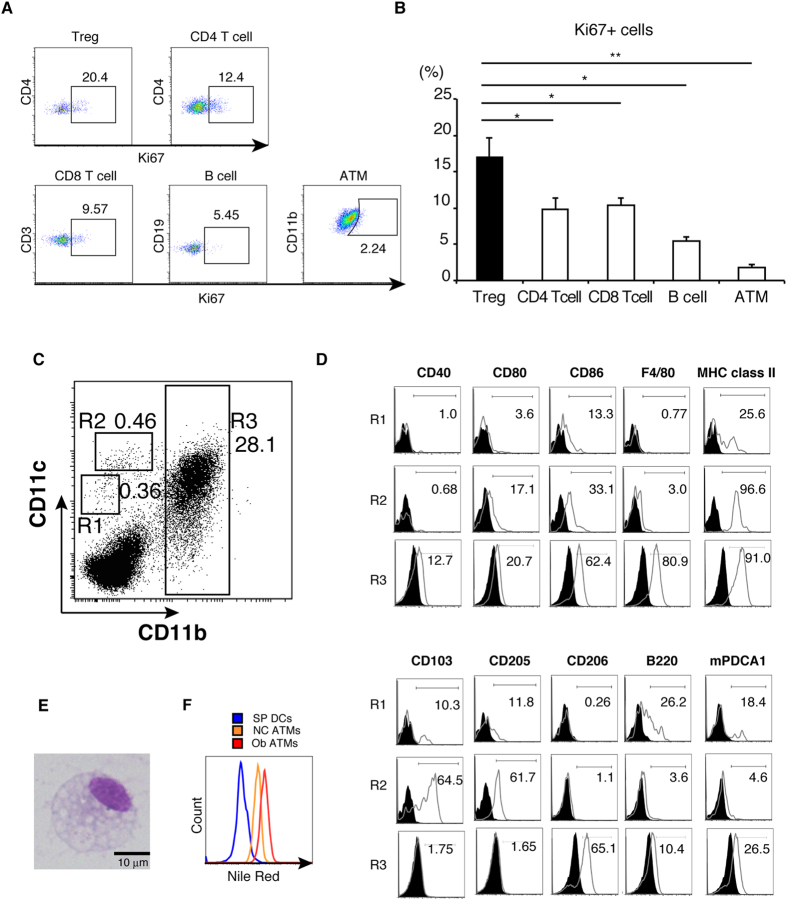
Local proliferation of adipose tissue Tregs and characterization of adipose tissue APC. (**A**) FACS analysis of Ki67^+^ cells in adipose tissue lymphocytes (CD3^+^ CD4^+^ cells (CD4 T cells), CD3^+^ CD8^+^ cells (CD8 T cell), B220^+^ CD19^+^ cells (B cell), CD3^+^ CD4^+^ FOXP3^+^ cells (Treg)) and F4/80+CD11b^+^ cells (ATMs). (**B**) The proportion of Ki67^+^ cells in each population calculated from results in (**A**). Data are mean ± SEM of three independent experiments. (**C**) Low-density leukocytes from the SVF were stained for CD11b, CD11c and F4/80 followed by flow cytometric analysis. (**D**) Low-density leukocytes from the SVF were stained for CD11b, CD11c, F4/80 and the indicated antibodies. (**E**) The R3 subset was sorted, and stained by the Pappenheim method, followed by microscopic analysis. (**F**) SPDCs and low-density cells of SVF from C57BL6/J mice, and ob/ob mice were stained with Nile red in the presence of CD11b, CD11c and F4/80 antibodies. Data are representative of two independent experiments.

Large numbers of Tregs are present in WAT. One possible explanation of this phenomenon is a proliferation of Treg in WAT. First, the intensity of Ki67, a cell proliferation marker, was measured in immune cells in adipose tissues. The expression of Ki67 was the highest in CD3^+^CD4^+^FOXP3^+^ Tregs among immune cells, including CD3^+^CD4^+^FOXP3^−^ T cells, CD3^+^CD8^+^ T cells, CD19^+^B220^+^ B cells, and F4/80^+^CD11b^+^ macrophages (Fig. 1A,B). This result suggested that antigen presenting cell (APC) seem to drive the proliferation of Tregs in adipose tissues. Next, we analyzed the characteristics of APCs in low-density leukocytes from the SVF based on CD11c and CD11b expression over a wide expression range^13^. Among these cells, at least three subsets of cells were recognized based on the CD11c/CD11b expression patterns: CD11c^int^CD11b^low^ (R1), CD11c ^high^ CD11b^low^ (R2), and CD11b ^high^ (R3) (Fig. 1C). R3 was the most abundant fraction, and flow cytometric analysis of the co-stimulatory molecules and surface antigens showed the expression of several DC-related molecules (CD40) and several important molecules for antigen presentation (MHC class II, CD80 and CD86) (Fig. 1D). In addition, R3 also expressed plasmacytoid DC-related molecule mPDCA1 as well as macrophage-related molecule (CD11b and F4/80), and CD206, a marker of M2 macrophages (Fig. 1D). With regard to the other two fractions, R1 expressed MHC class II, CD103, CD205, B220, and mPDCA1 (Fig. 1D), which is in agreement with the characteristics of plasmacytoid dendritic cells (pDCs). On the other hand, R2 overexpressed CD11c, MHC class II, CD103, and CD205. The expression of these co-stimulatory molecules was consistent with the conventional features of DC (Fig. 1D). Based on these results, we used the F4/80^+^ R3 subset as ATMs.

To further characterize the ATMs, we analyzed the morphology of R3. Microscopic analysis using Pappenheim staining showed that R3 contained droplets or cytoplasmic vacuoles characteristic of macrophages (Fig. 1E). Furthermore, Nile red staining showed more neutral lipid content in ATMs from ob/ob mice (ob/ob ATMs) than those from C57BL6/J mice (NC ATMs) (Fig. 1F).

### Induction of Tregs by ATMs

**Figure 2:**
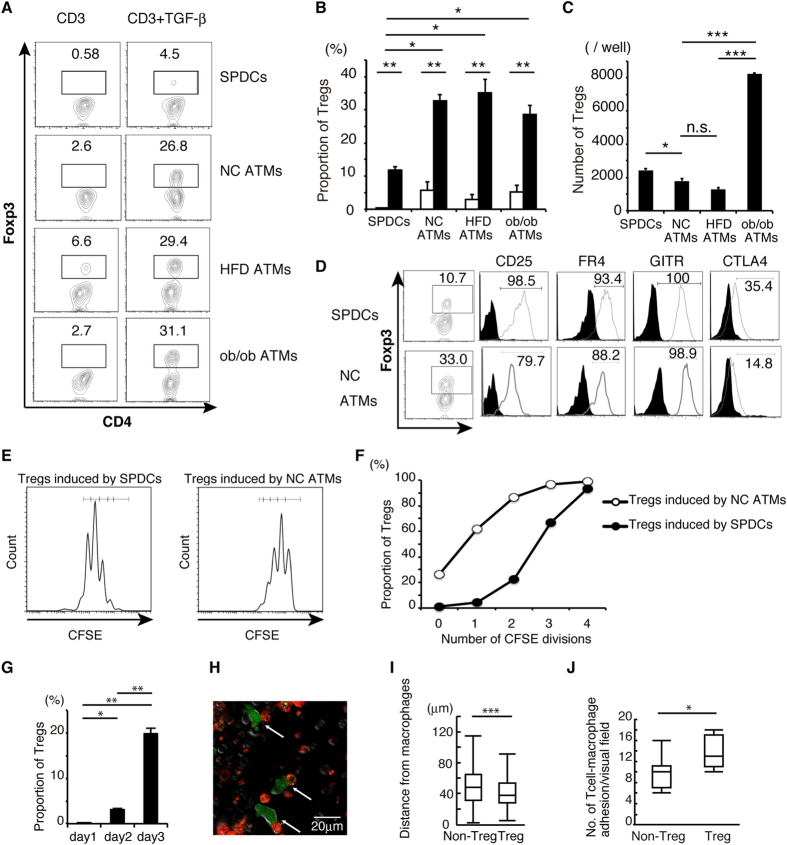
ATMs are closely related to Tregs to induce FOXP3^+^ regulatory T cells *in vitro.* (**A**) FACS analysis of T cells co-cultured with ATMs or SPDCs from NC, HFD, and ob/ob mice. FOXP3^+^ T cells were determined as Tregs. (**B**) The proportion of Tregs calculated from results in (A). Data are mean ± SEM of three independent experiments. (**C**) Numbers of Tregs induced by ATMs or SPDCs from NC, HFD, and ob/ob mice were calculated. (**D**) CD25, FR4, GITR and CTLA4 expression in Tregs induced *in vitro* by SPDCs and ATMs. (**E**) CFSE labeled non Treg T cells were cocultured with SPDCs or ATMs. CFSE fluorescence of induced Tregs was analyzed on day 3. (**F**) The integrated proportion of proliferated Tregs among total induced Tregs is plotted. (**G**) The proportion of FOXP3-EGFP positive regulatory T cells was determined by confocal microscopy during *in vitro* Treg induction using NC ATMs. (**H**) Dynamics of NC ATMs (red) stained by PE-CD11b antibody and FOXP3-EGFP positive regulatory T cells (green) were visualized by confocal microscopy. (**I**) Box-and-whisker plots of distance between macrophages and Tregs or non-Treg T cells on day 3 (n=149 for Tregs and n=940 for non Treg T cells). (**J**) Box-and-whisker plots of the number of non-Treg T cells and Tregs attached to macrophages for more than 6 min (n=96 for Tregs and n=67 for non Treg T cells). In the box-and-whisker plots, lines within the boxes represent median values; the upper and lower lines of the boxes represent the 25th and 75th percentiles, respectively; and the upper and lower bars outside the boxes represent the 90th and 10th percentiles, respectively. Samples were measured in triplicate. *Data pooled from two independent experiments*. For (B, C, G, I, J): *P<0.05, **P<0.01, ***P<0.001.

Tregs are abundant in adipose tissues accounting for almost 40% of T cells^10^, and proliferate in adipose tissues (Fig. 1A,B). Considering that ATMs are the most abundant APCs (Fig. 1C), we hypothesized that ATMs might be potent inducers of Tregs. To test this hypothesis, ATMs, SPDCs, and CD4^+^CD25^−^CD62L^+^CD44^−^ (non Treg) T cells were purified using FACS. Subsequently, non Treg T cells were stimulated with ATMs or SPDCs plus anti-CD3 mAb, with or without TGF-β. After 6-day culture, SPDCs induced FOXP3^+^ Tregs (without TGF-β: 0.58%, with TGF-β: 4.5%) (Fig. 2A). Surprisingly, the proportion of FOXP3^+^ Tregs induced by ATMs was higher than by SPDCs in the presence of TGF-β (NC ATMs: 26.8%, HFD ATMs: 29.4%, ob/ob ATMs: 31.1%) (Fig. 2A). SPDCs and ATMs induced Tregs with quite low efficiency in the absence of TGF-β, indicating that this effect was dependent on TGF-β. The proportion of induced Tregs was higher in three ATMs than SPDCs (Fig. 2A,B), however, the number of Tregs was much higher in ob/ob ATMs (Fig. 2C). SPDC increased the number of Tregs more than NC or HFD ATMs (Fig. 2C). These results indicated that SPDCs and ob/ob ATMs increased both Tregs and other T lymphocytes, whereas, NC and HFD ATMs efficiently increased Tregs compared to other T lymphocyte.

To further characterize Treg induction *in vitro*, we analyzed CD25, folate receptor (FR)-4, glucocorticoid-induced tumor necrosis factor receptor (GITR), and cytotoxic T-lymphocyte-associated protein (CTLA) 4, which are hallmarks of Treg^5^,^14^,^15^,^16^. In Tregs induced by SPDCs and NC ATMs, all Treg markers, such as CD25, FR4 and GITR, were highly upregulated to levels similar to those induced by SPDCs (Fig. 2D). CTLA4 induction was relatively low in Tregs induced by ATMs (Fig. 2D). Next, number of CFSE (carboxyfluorescein diacetate succinimidyl ester) divisions were measured during Treg induction *in vitro*. In Tregs induced by ATMs, number of CFSE division was lower than that in Tregs induced by SPDCs (Fig. 2E,F). These results indicated that ATMs converted non-Treg T cells into *de novo* Tregs more efficiently than SPDCs.

To further confirm the induction of Tregs by ATMs, CD3^+^CD4^+^FOXP3^−^ (non Treg) T cells were collected from FOXP3-EGFP mice, which co-express EGFP and FOXP3 under the control of endogenous FOXP3 promoter. These non-Treg T cells were cocultured with ATMs from control mice, and examined by time-lapse confocal microscopy. In this system, conversion of Tregs was reflected by GFP fluorescence. The frequency GFP-positive cells increased with time (Fig. 2G), indicating that coculture with ATMs converted non-Treg T cells into Tregs. During microscopic observation, firm adhesion was observed between Tregs and ATMs (Fig. 2H, green cells: Tregs, red cells: ATMs). Previously, Cahalan *et al.* reported that contacts between Tregs and DCs last longer than those between conventional T cells and DCs, and this interaction is mediated by CTLA4^17^. Similarly to this report, distance analysis indicated that the distance between ATMs and Tregs was significantly shorter than that between ATMs and non-Treg T cells (Fig. 2I, supplementary Figure 1A,B). Moreover, adhesion between ATMs and Tregs was more frequent than that between ATMs and non-Treg T cells (Fig. 2J). Considering that ATMs induced CTLA4 positive Tregs *in vitro* (Fig. 2D), interaction between Tregs and ATMs should last longer than non-Tregs. Collectively, these results indicate that ATMs had potency to convert non-Treg T cells into Tregs *in vitro*.

### *In vivo* and *in vitro* characterization of Tregs

**Figure 3:**
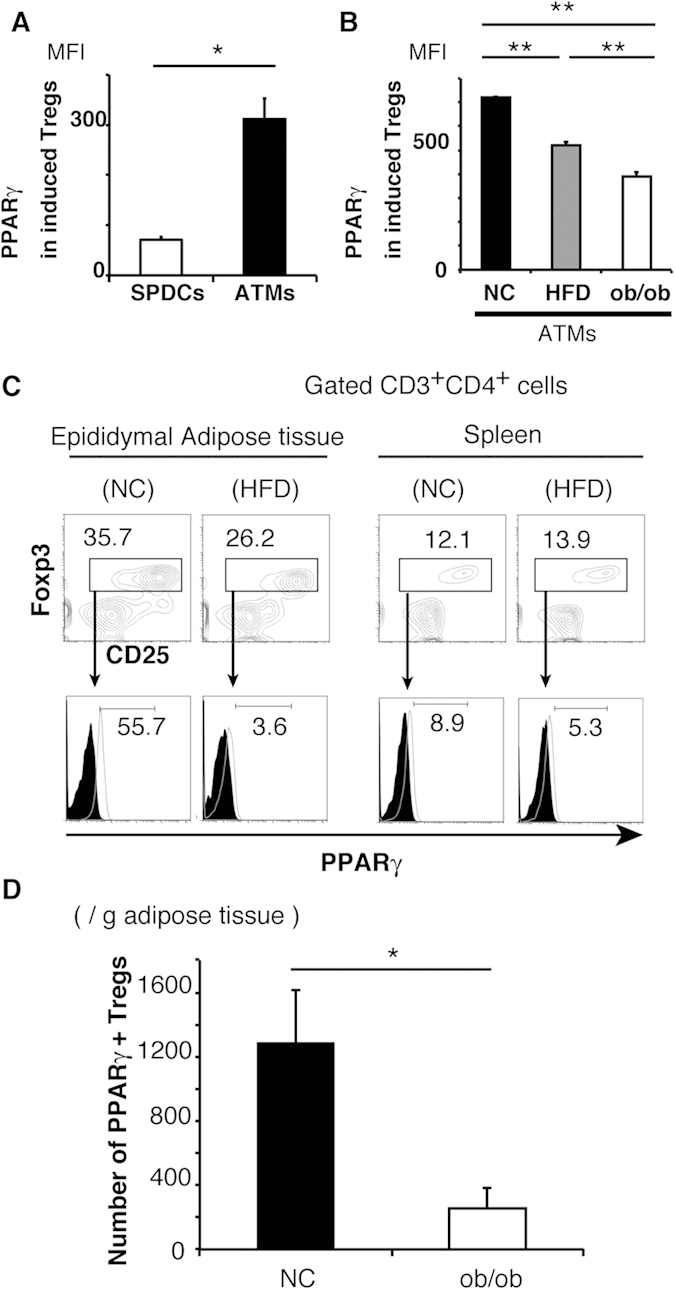
NC-fed but not HFD mice-derived ATMs prompt PPARγ-high Treg differentiation. (**A**) PPARγ expression in Tregs induced by NC SPDCs and NC ATMs. (**B**) PPARγ expression in Tregs induced by NC ATMs, HFD ATMs, and ob/ob ATMs. (**C**) Splenic T cells and adipose T cells isolated from NC and HFD mice were stained with CD25, FOXP3, and PPARγ. The proportion of PPARγ-high FOXP3^+^ Tregs was analyzed by FACS. (**D**) Numbers of PPARγ-high Tregs in NC and ob/ob mice were counted by FACS. Data are representative of at least three independent experiments (**A**–**C**). Data are mean ± SEM. For (**A**,**B**,**D**): *P < 0.05, **P < 0.01.

Since Tregs from adipose tissues of NC mice specifically express PPARγ^10^, we further characterized Tregs induction *in vitro* by measuring PPARγ expression. PPARγ expression level was higher in Tregs induced by NC ATMs than those induced by SPDCs of NC (Fig. 3A). Moreover, PPARγ expression level was significantly higher in Tregs induced by NC ATMs than those by HFD ATMs (Fig. 3B). Furthermore, PPARγ expression was lower in Tregs induced by ob/ob ATMs than those by HFD ATMs (Fig. 3B), indicating that lean ATMs induced PPARγ-high Tregs, whereas obese ATMs induced PPARγ-low Tregs *in vitro*.

To investigate the impact of obesity on ATMs, we performed gene expression microarray analysis of ATMs derived from NC ATMs and ob/ob ATMs (supplementary Figure 2). Ob/ob ATMs showed significantly higher expressions of inflammatory cytokines, such asIL-1β and TNF-α, and lower expression of Th2 cytokine: IL-4 than NC ATMs. In addition, Sema4a expression was significantly lower in ob/ob ATMs than NC ATMs. There were no significant differences in expressions of IL-33 or TGFβ1, which were cytokines associating Treg induction^18^,^19^. IL-1β is reported to convert human Treg into Th17 cells^20^. TNF-α induces depohsphorylation of Foxp3, and inhibits Treg function^21^. Sema4a binds to nrp1 in Tregs, and potentiate Treg function and survival^22^. These results indicated that differences between obese and NC ATMs might be derived from such changes in cytokine and surface molecule expressions.

Next, we characterized adipose Tregs *in vivo*. The proportion of Tregs was higher in adipose tissues than spleen, and higher in adipose tissues of NC than HFD mice (Fig. 3C; upper), as reported previously^10^. Under these conditions, PPARγ expression was markedly higher in adipose NC Treg than HFD (NC: 55.7%, HFD: 3.6%) (Fig. 3C; lower). PPARγ expression level was lower in Tregs from the spleen than those from adipose tissue of NC, and the expression level in the spleen was not different between NC and HFD (NC: 8.9%, HFD: 5.3%) (Fig. 3C; lower). Number of PPARγ-high Tregs were significantly higher in adipose tissues of NC than ob/ob mice (Fig. 3D). Collectively, based on the ability of NC ATMs to induce PPARγ-high Tregs *in vitro*, PPARγ-high Tregs were abundant in adipose tissues of NC mice *in vivo*.

### Effects of macrophage ablation by clodronate on adipose Tregs

**Figure 4:**
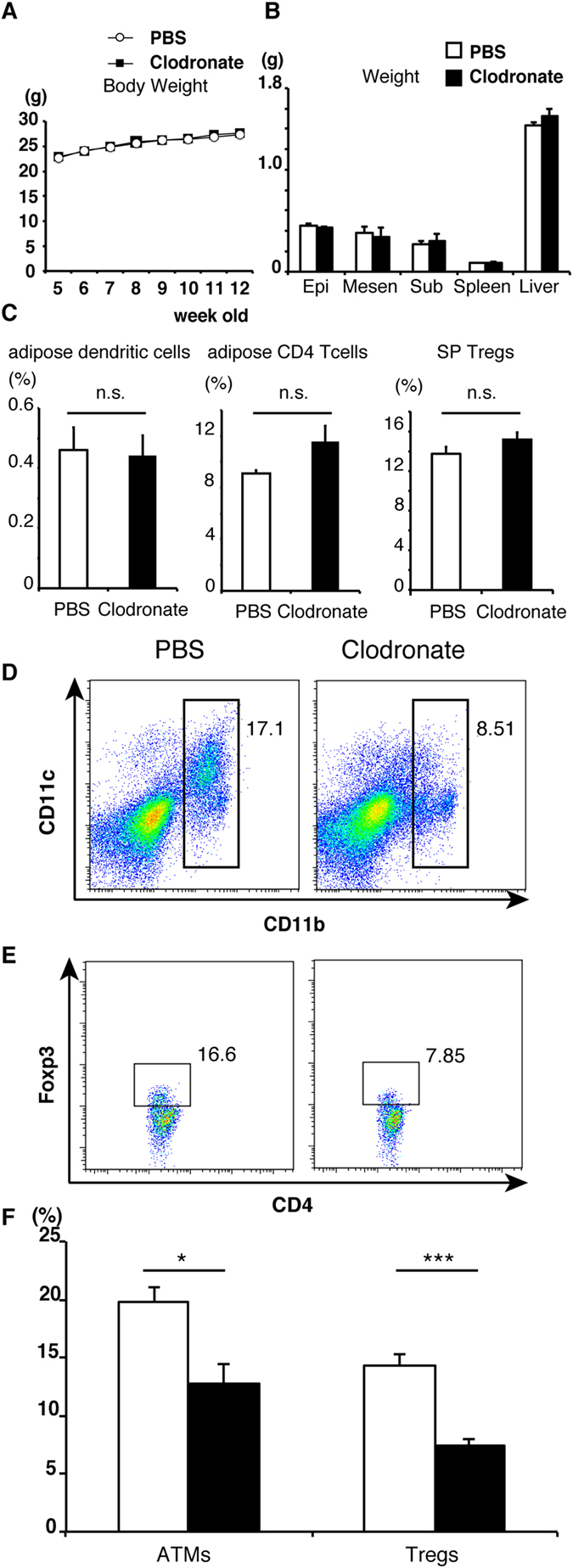
Changes in proportion of Treg after clodronate ablation of macrophages. (**A**) Serial changes in body weight in each group (n = 3 for clodronate treated group and n = 9 for PBS treated group). (**B**) Summary of organ weight (epididymal fat, mesenteric fat, subcutaneous fat, spleen and liver) in each group. Data are mean ± SEM of three independent experiments (**A**,**B**). The proportions of adipose dendritic cells ((**C**); left), adipose CD4 T cells ((**C**)); middle), splenic Tregs ((**C**); right), ATMs (**D**) and adipose Tregs (**E**) obtained from NC-fed mice were analyzed by FACS. *Right*: clodronate-treated group, *left*: PBS-treated group. (**F**) The proportion of ATMs and adipose Tregs in each group calculated from results in (**D**,**E**). Data are mean ± SEM of three independent experiments. For (**F**): *P < 0.05, ***P < 0.001.

Feng *et al.*^23^,^24^ reported that intraperitoneal injection of clodronate-liposomes reduced the number of ATMs in diet-induced obese mice. Using the same experimental protocol, NC mice were treated with PBS-liposome or clodronate-liposome for 8 weeks. No significant differences were observed in the time course of body weight and organ weights (epididymal fat, mesenteric fat, subcutaneous fat, spleen and liver) between PBS- and clodronate-liposome injected groups (Fig. 4A,B). Clodronate-liposome did not alter the proportion of dendritic cells, or CD4^+^ T cells in adipose tissue, and splenic Tregs (Fig. 4C), but significantly reduced ATMs (Fig. 4D,F). Under this condition, adipose Tregs were significantly decreased (Fig. 4E,F).

### Induction of Tregs by ATMs *in vivo*

**Figure 5:**
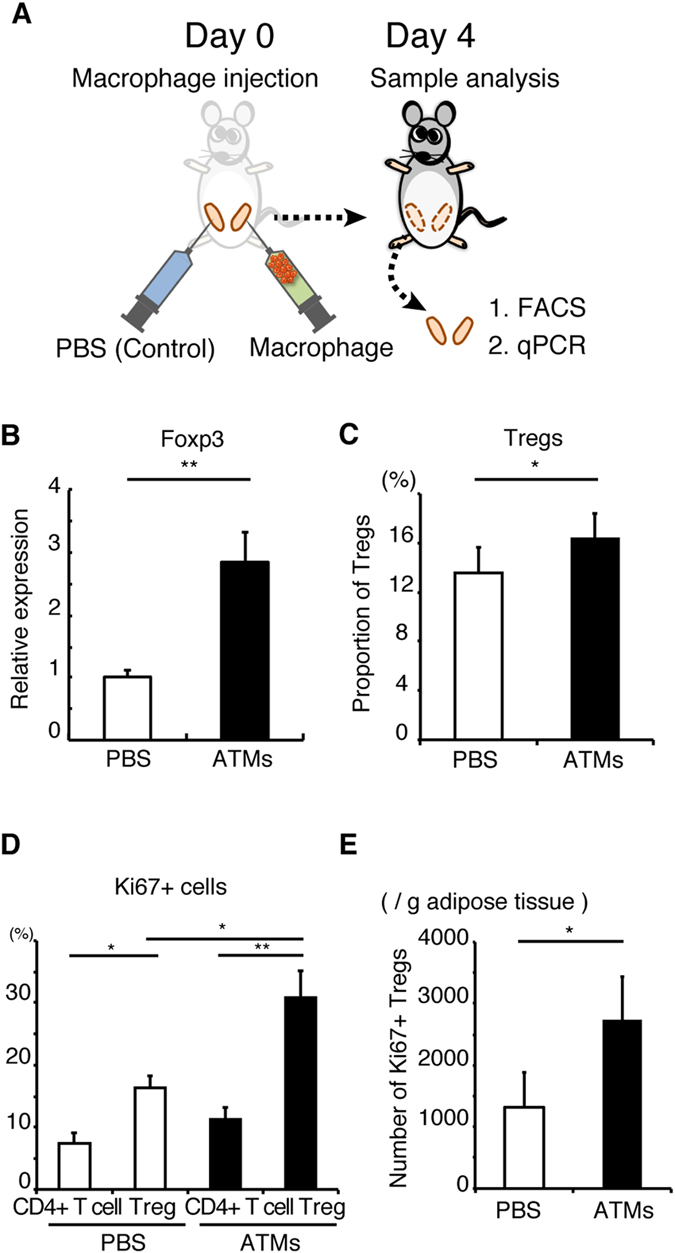
Effects of ATMs on local proliferation of adipose Tregs. (**A**) ATMs were injected into one side of epididymal adipose tissues in C57BL6/J mice. Four days after the injection, epididymal adipose tissues were collected and used for FACS analysis. (**B**) Relative expression of FOXP3 in ATMs injected adipose tissues. Data are mean ± SEM of n = 4 per group. (**C**) The proportion of adipose Tregs in ATMs injected adipose tissues. (**D**) The proportion of Ki67+ cells in adipose Tregs and CD3 + CD4 + FOXP3- T cells (CD4^+^ T cells). (**E**) Numbers of Ki67+ Tregs was analyzed by FACS. Data are mean ± SEM of at least two independent experiments (**C**–**E**). For (**B**–**D**): *P < 0.05, **P < 0.01.

Next, to investigate the differentiation of Tregs in adipose tissues, isolated ATMs or PBS was injected into each side of epididymal WAT in living mice, followed by analysis of WAT after 4 days (Fig. 5A). FOXP3 expression and the proportion of Treg were significantly higher in WAT injected with ATMs than with PBS (Fig. 5B,C). Furthermore, the expression of Ki67 was higher in Tregs than other CD4^+^ T cells in each WAT (Fig. 5D). Moreover, Ki67 expression was higher in Tregs from ATM-injected WAT than PBS-injected WAT (Fig. 5D). The number of Ki67 + Tregs was significantly higher in ATM-injected WAT than PBS-injected WAT (Fig. 5E). Considered together, ATMs induce differentiation and proliferation of Tregs *in vivo*.

To rescue the Treg number in WAT of obese mice, isolated NC ATMs or PBS was injected into each side of epididymal WAT in ob/ob mice. The proportion of Tregs was not different between PBS-injected and ATM injected WAT, and the number of Tregs showed tendency to decrease in ATM-injected WAT (supplementary Figure 3A, B). These results indicated that NC ATMs could not rescue Treg cell number in ob/ob mice.

### Effects of adiponectin deficiency on ATMs and Tregs

**Figure 6:**
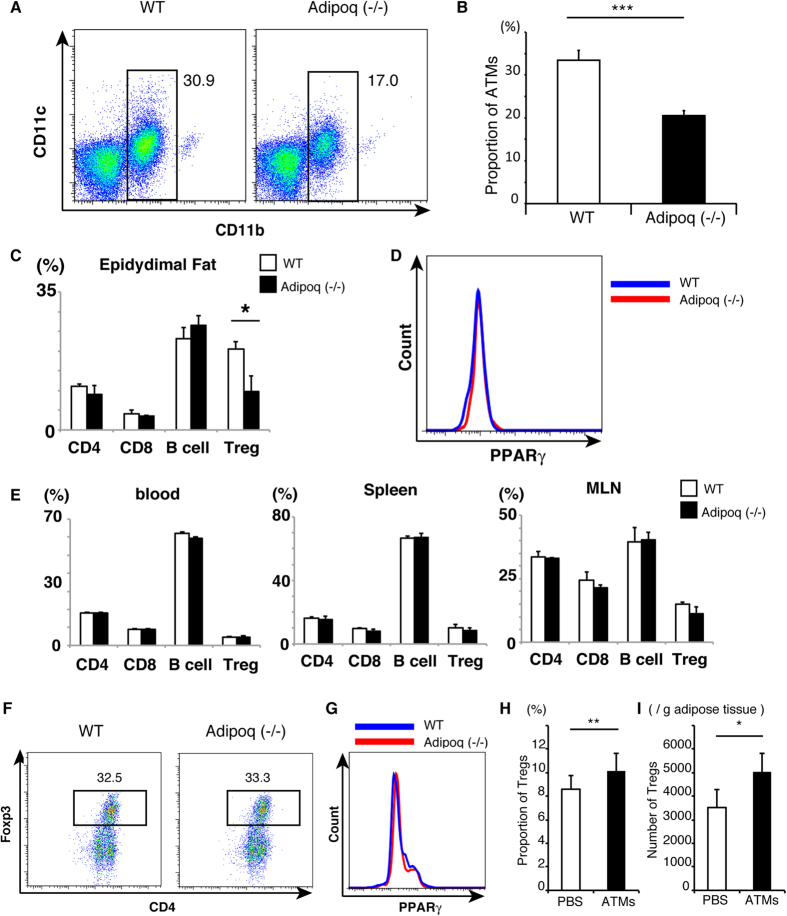
Effects of adiponectin deficiency on ATMs and Tregs. (**A**) FACS analysis of ATMs obtained from 10 to 14-week-old wild-type (WT) and adiponectin deficient mice [Adipoq (−/−)]. (**B**) The proportion of ATMs in WT and Adipoq (−/−) mice calculated from results in (**A**). Data are mean ± SEM (n = 7 for WT, n = 3 for Adipoq (−/−)). (**C**) The proportion of lymphocytes (CD3^+^CD4^+^ T cells (CD4), CD3^+^CD8^+^ T cells (CD8), B220^+^CD19^+^ B cells (B cells), and CD3^+^CD4^+^FOXP3^+^ cells (Treg)) from epididymal adipose tissues of WT mice (open bars) and Adipoq (−/−) mice (closed bars). (**D**) Comparison of PPARγ expression in adipose Tregs of WT and Adipoq (−/−) mice. (**E**) The proportion of lymphocytes (CD4, CD8 T, B cells and Tregs) in blood, Spleen, and mesenteric lymph nodes (MLN) of WT mice (open bars) and Adipoq (−/−) mice (closed bars). Data are mean ± SEM (n = 3 per group). (**F**) Purified ATMs from WT and Adipoq (−/−) mice were cocultured with non-Treg T cells in the presence of TGF-β. The proportion of CD4^+^FOXP3^+^ regulatory T cells was determined after 6-day culture. (**G**) PPARγ expression in Tregs induced by ATMs from WT (blue) and Adipoq (−/−) mice (red). Data are representative of two independent experiments. (**H**) Isolated ATMs were injected into one side of epididymal adipose tissue of Adipoq (−/−) mice as shown in Fig. 5 (**A**). The proportion of adipose Tregs were analyzed by FACS. (**I**) The Numbers of adipose Tregs were calculated by FACS. Data are mean ± SEM of four independent experiments (**H**,**I**). For (**B**,**C**,**H**,**I**): *P < 0.05, **P < 0.01 and ***P < 0.001.

Next, we estimated the proportion of ATMs and adipose lymphocytes in adiponectin-deficient (KO) mice [Adipoq (−/−)]. As shown in Fig. 6A, the proportion of ATMs in adipose tissues was lower in Adipoq (−/−) mice than WT mice (Fig. 6A,B), though adipose DCs were not altered in Adipoq (−/−) (supplementary Figure 4). The proportion of Tregs in epididymal fat, but not CD4, CD8 T cells, and B cells, was significantly lower in Adipoq (−/−) than WT (Fig. 6C). On the other hand, PPARγ expression in adipose Tregs was not different between WT and Adipoq (−/−) (Fig. 6D). The proportion of Tregs showed no differences between Adipoq (−/−) and WT mice in blood, spleen, or MLN (Fig. 6E).

To further investigate the mechanism, Tregs were induced by ATMs from WT mice and Adipoq (−/−) mice *in vitro*. Coculture of the same number of ATMs with non-Treg T cells resulted in the induction of similar proportions of Tregs in WT and Adipoq (−/−) (Fig. 6F), and PPARγ expression in Tregs was not different between those induced by ATMs from WT or Adipoq (−/−) (Fig. 6G).

To confirm the effect of ATMs, NC ATMs were introduced into epididymal WAT of Adipoq (−/−) mice. The rate and number of Tregs were significantly increased in ATM-injected WAT than PBS (Fig. 6H,I). These results suggest that the small proportion of ATMs in Adipoq (−/−) mice should be related to the small proportion of adipose Tregs.

## Discussion

In the present study, we characterized ATMs from NC and obese mice. These cells differentially promoted the induction of Tregs. ATMs of NC induced PPARγ-high Tregs while ATMs of obese mice induced PPARγ-low Tregs. Moreover, we demonstrated that PPARγ expression was markedly reduced in adipose Treg of HFD-fed obese mice compared with control mice. In agreement with these results, ATMs of HFD-fed mice induced Tregs with lower PPARγ expression than control mice *in vitro*. Furthermore, ATMs of severe obese ob/ob mice induced Tregs with further lower PPARγ expression than HFD-fed mice *in vitro*. Consequently, the severity of obesity correlated with the low proportion of adipose Tregs and their PPARγ expression *in vivo*, and was linked with the diminished ability of ATMs to induce Tregs with PPARγ expression *in vitro*.

To further assess the ability of ATMs to induce Tregs *in vivo*, we performed both ablation and adoptive transfer of macrophages in epididymal WAT. To deplete ATMs *in vivo*, we used a chemical reagent that produced local ablation of macrophages in epididymal WAT. In this regard, intraperitoneal injection of clodronate-liposome is reported to reduce the number of macrophages in fat depots located within the peritoneal cavity, particularly in epididymal WAT^23^,^24^. In the present study, clodronate-liposome significantly reduced ATMs without reducing adipose dendritic cells, as well as significantly reduced the number of adipose Treg in steady state conditions. The results suggest a major role for ATMs in the maintenance of adipose Tregs. Inversely, injection of ATMs resulted in significant increase in the proportion and the number of Ki67-expressing adipose Tregs. The result suggests that ATMs play a role in inducing differentiation and proliferation of Tregs *in vivo*.

Injection of NC ATMs increased the proportion of Tregs in WAT of NC mice, but not in WAT of ob/ob mice. These results indicated that Treg induction was influenced by environmental state. The expression of IL-6 is increased in adipose tissues of obese mice, and its level in adipose tissue correlates with insulin resistance^25^. Previous studies indicated that in the absence of proinflammatory cytokines, such as IL-6 or IL-21, TGF-β induced differentiation of naive T cells to Tregs, whereas in the presence of IL-6, naive T cells differentiated into proinflammatory Th17 cells^18^. Actually, we confirmed that IL-6 abrogated the efficient induction of Tregs by ATMs (supplementary Figure 3C, D). Collectively, inflammatory state should be partly account for the low efficiency of Treg induction in WAT of obese mice.

Adiponectin is a protein secreted abundantly by adipocytes^11^. Serum adiponectin levels correlate inversely with anthropometric indices and insulin resistance in obese subjects^26^. Furthermore, adiponectin promotes polarization of anti-inflammatory macrophages^27^. Previous studies also showed accumulation of adiponectin protein in immune cells-containing SVF, in adipose tissues^12^. However, the effects of adiponectin on adipose tissue inflammation and ATMs remain unclear. The present study showed that the effect of adiponectin on adipose tissue immune cells includes the maintenance of ATM number without affecting FOXP3 and PPARγ induction. The proportions of ATMs and Tregs were lower in adipose tissues of Adipoq (−/−) compared with WT mice. The ability of ATMs to induce PPARγ-expressing Tregs in both WT and Adipoq (−/−) mice was similar *in vitro*. Consequently, the low number of Tregs is related to the small number of ATMs in Adipoq (−/−) mice. Adiponectin deficiency leads to diet-induced insulin resistance^11^, and Treg depletion worsens insulin resistance in obese mice^28^. Considered together, the small numbers of ATMs and Tregs may partly contribute to the development of insulin resistance in Adipoq (−/−) mice.

Recently, Mathis *et al.*^29^ reported that the adipose Treg compartment is seeded from thymocytes, and that the accumulation of Treg depends on APCs, including DCs and ATMs^29^. Our results demonstrate that ATMs, but not DCs, have a large impact on the proportion of adipose Tregs. In addition, our experiments clearly demonstrated the potency of ATMs in converting non-Treg T cells into Tregs *in vitro*. Moreover, in Tregs induced by ATMs, the number of cell division was smaller than those by SPDCs, indicating that ATMs generated *de novo* Tregs more efficiently than SPDCs. These results suggest that ATMs could induce *de novo* generation of Tregs in adipose tissues.

In summary, the present study demonstrated that ATMs of control mice promoted the differentiation of PPARγ-high Tregs, whereas ATMs of obese mice triggered PPARγ-low Tregs. In obese adipose tissues, the diminished capacity of ATMs to induce PPARγ-high Tregs and low adiponectin is likely linked to the low number of Tregs.

## Research Design and Methods

### Animals

The experimental protocol was approved by the Ethics Review Committee for Animal Experimentation of Osaka University, Graduate School of Medicine. All animal experiments were carried out in accordance with the Institutional Animal Care and Use Committee Guidelines of Osaka University. Male C57BL6/J and ob/ob mice were purchased from Charles River Japan (Yokohama, Japan) and used in experiments at 11–16 weeks of age. Control mice were fed normal chow (NC). For diet-induced obesity, 5-week-old C57BL6/J mice were fed high-fat/high-sucrose diet (HFD) for 9–12 weeks and used at 14–17 weeks of age. FOXP3 bicistronic reporter knock-in mice expressing EGFP were kindly provided by Dr. Kiyoshi Takeda (Osaka University, Japan). Adiponectin-deficient mice were generated as described previously^11^ and used at 10–14 weeks of age. All mice were maintained under specific pathogen-free conditions and had free access to water and chow.

### Isolation of ATMs

ATMs were collected as described previously^30^. The spleen was digested with 400 Mandl units/ml collagenase D (Roche) and 10 μg/ml DNase I (Roche) under continuous stirring at 37 °C for 45 min. The cell suspensions were spun through 17.5% Accudenz (Accurate Chemical and Scientific Corporation, Westbury, NY) solution to obtain a population rich in macrophages and DCs. Cells were sorted by FACSAria II (BD Biosciences, Bedford, MA), and the purity of the obtained macrophages was >95%.

### Flow cytometry

Fluorescence-activated cell sorting (FACS) analysis was carried out as described previously^30^. Briefly, cells in the SVF were suspended in FACS buffer and incubated with anti-mouse CD16/CD32 (93; Biolegend, San Diego, CA) for 15 min. Then, the cells were rinsed and resuspended in FACS buffer and stained with anti-CD11c (HL3; Biolegend) and anti-CD11b (M1/70: Biolegend) antibodies for DC and macrophages, respectively. The expression of co-stimulatory molecules was determined using mAbs to CD80 (16-10A1; BD), CD86 (GL1; BD), CD40 (3/23; BD), and MHC class II (M5/114.15.2; BD). The expression of other surface antigens was analyzed by using mAbs to F4/80 (BM8; Biolegend), CD103 (M290; BD), CD205 (NLDC145; MACS), CD206 (MR5D3; AbD Serotec), mPDCA1 (JF05-1C2.4.1:MACS), and B220 (RA3-6B2; Biolegend). To detect lipid, cells were first stained with 1 μg/ml Nile red (Wako Pure Chemicals, Osaka, Japan) for 15 min, and then analyzed with FACSVerse (BD Biosciences). The expressions of Treg related molecules were determined by using mAbs to CD25 (PC61; Biolegend), FR4 (12A5; Biolegend), CTLA4 (UC10-4B9; Biolegend) and GITR (DTA-1; Biolegend).

### Intracellular staining

Intracellular staining of FOXP3 was conducted by using FOXP3/Transcription factor staining buffer set according to the instructions provided by the manufacturer (eBioscience, San Diego, CA). For PPARγ or Ki67 staining, polyclonal anti- PPARγ antibody (PA5-25757; Thermo Scientific) or anti-Ki67 antibody (Biolegend) was added with FOXP3-antibody (FJK-16; eBioscience), followed by the addition of secondary antibody (406410; Biolegend).

### T cell conversion assay

2 × 10^4^ CD4^+^CD25^−^CD62L^+^CD44^−^ non Treg T cells from WT mice or CD4^+^FOXP3^−^ non Treg T cells from FOXP3-EGFP mice were cultured with 4 × 10^3^ purified ATMs or splenic dendritic cells (SPDCs) in 200 μl of complete medium with soluble 1 μg/ml anti-CD3e mAb (1452C-11; Biolegend) and human 2 ng/ml rTGF-β (R&D systems, Minneapolis, MN) for 6 days. On day 6, the proportion of Tregs and PPARγ expression was determined by FACS.

### Proliferation assay

CD3^+^CD4^+^Foxp3^−^ splenocytes from control mice were labeled with 1.25 μM CFSE (Biolegend) in PBS. CFSE labeled T cells were cultured with DCs or ATMs at 1:1 ratio and stimulated with 1 μg/ml anti-CD3e mAb for 72 hours. Cellular proliferation was calculated by CFSE dilution using FACS Verse.

### Confocal time-lapse imaging

PE-CD11b labeled ATMs and CD4^+^FOXP3^−^ T cells from FOXP3-EGFP mice were co-cultured on glass bottom plate (Greiner Bio One, Stonehouse, UK). Time-lapse confocal microscopy was performed using A1 confocal microscope (Nikon), followed by cell position analysis using Imaris software (Bitplane, Zurich, Switzerland). Using position data, contour plots of ATMs distribution were generated by the JMP software (SAS Institute, Cary, NC). After selection of eight local maximum points containing abundant ATMs, the minimum distance between the point and each T cell was measured as the distance from macrophages (supplementary Figure 5A, BM).

### Clodronate depletion from macrophages

Clodronate liposomes were purchased from clodronateliposomes.org (Vrije Universiteit, Netherlands) at concentration of 1 mg/ml, and prepared as described previously^23^,^24^. Five-week-old mice were fed normal chow and concomitantly treated with 150 μL liposome-clodronate or an equivalent volume of liposomes containing phosphate buffered saline (PBS) once per week for 8 weeks.

### Quantitative real-time PCR

Total RNA was prepared from freshly isolated cells using RNAprotect Cell Reagent (Qiagen) using the protocol supplied by the manufacturer. RNA purification was performed using RNeasy microkit (Qiagen) following the instructions provided by the manufacturer. The cDNA was synthesized using the Transcriptor First Strand cDNA Synthesis Kit (Roche, Indianapolis, IL). Real-time PCR was performed on the LightCycler system (FastStart DNA Master SYBR Green I, Roche) according to the protocol provided by the manufacturer.

### Macrophage adoptive transfer

9 × 10^5^ F4/80^+^CD11b^+^ macrophages derived from adipose tissues were injected into one side of adipose tissues with PBS as vehicle. Four days after injection, adipose tissues were collected. FOXP3 expression was quantified by quantitative polymerase chain reaction (qPCR) and the proportion of Ki67^+^ Tregs was analyzed by flow cytometry.

### Microarray analysis

The macrophage subsets were sorted from epididymal adipose tissues of male C57BL6/J and ob/ob mice at 11 weeks of age, based on their expression of CD11b, CD11c and F4/80. RNA was extracted with RNeasy micro kit. Gene expression profiling was performed with Mouse Gene Expression 8 × 60 K Microarray (Agilent, Santa Clara, CA) by Takara Bio Inc (Tokyo, Japan). Cyanine-3 (labeled cRNA was prepared from 0.1 μg Total RNA using the Low Input Quick Amp Labeling Kit (Agilent). Fragmentation, hybridization, and washing steps were also carried out as recommended by the manufacturer (Agilent). Slides were scanned on the Agilent SureScan Microarray Scanner G2600D using one color scan setting for 8 × 60 K array slides. The scanned images were analyzed with Feature Extraction Software 11.5.1.1 (Agilent) using default parameters to obtain processed signal intensities.

### Statistical analysis

All data were expressed as mean ± SEM. Differences between two groups were examined for statistical significance by the Student’s t-test. A *P* value < 0.05 denoted the presence of a statistically significant difference.

## Additional Information

**How to cite this article**: Onodera, T. *et al.* Adipose tissue macrophages induce PPARγ-high FOXP3^+^ regulatory T cells. *Sci. Rep.* **5**, 16801; doi: 10.1038/srep16801 (2015).

## Supplementary Material

Supplementary Information
