# Supplementary material for: Adipose tissue macrophages induce PPARγ-high FOXP3+ regulatory T cells
Source: Sci Rep. 2015 Nov 19;5:16801. doi: 10.1038/srep16801 (PMC4652162; doi:10.1038/srep16801)
Supplement: Supplementary Information [file srep16801-s1.pdf]

# Supplementary Information

## Adipose tissue macrophages induce PPARγ-high FOXP3$^+$ regulatory T cells

Toshiharu Onodera [a], Atsunori Fukuhara [a], Myoung Ho Jang [b], Jihoon Shin [a], Keita Aoi [c,d], Junichi Kikuta [c,d], Michio Otsuki [a], Masaru Ishii [c,d], Iichiro Shimomura [a]

[a] Department of Metabolic Medicine, Osaka University Graduate School of Medicine, 2-2, Yamamdaoka, Suita, Osaka, Japan

[b] Academy of Immunology and Microbiology (AIM), Institute for Basic Science (IBS), Pohang 790-784, Korea

[c] Department of Immunology and Cell Biology, Osaka University Graduate School of Medicine & Frontier Biosciences, WPI-Immunology Frontier Research Center, 2-2, Yamamdaoka, Suita, Osaka, Japan

[d] JST, CREST, 5 Sanban-cho, Chiyoda-ku, Tokyo, Japan

***Corresponding author:***Atsunori Fukuhara, MD, Department of Metabolic Medicine, Graduate School of Medicine, Osaka University, 2-2 Yamadaoka, Suita, Osaka 565-0871, JAPAN, Tel: +81-6-6879-3732, Fax: +81-6-6879-3739, E-mail: fukuhara@endmet.med.osaka-u.ac.jp

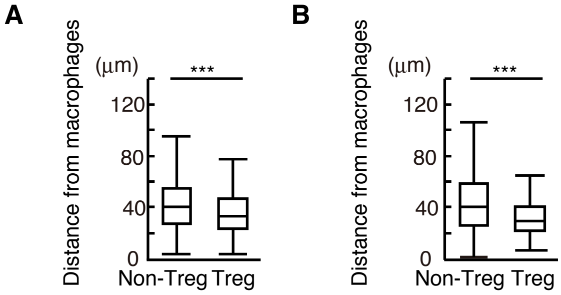


Supplementary figure 1. Distance from macrophages to Tregs or non-Treg T cells in independent two experiment.

(A) Box-and-whisker plots of distance between macrophages and Tregs or non-Treg T cells on day 3 (n=29 for Tregs and n=328 for non Treg T cells). (B) Box-and-whisker plots of distance between macrophages and Tregs or non-Treg T cells on day 3 (n=120 for Tregs and n=612 for non Treg T cells). Pooled data from these experiments are indicated in figure 2(H).

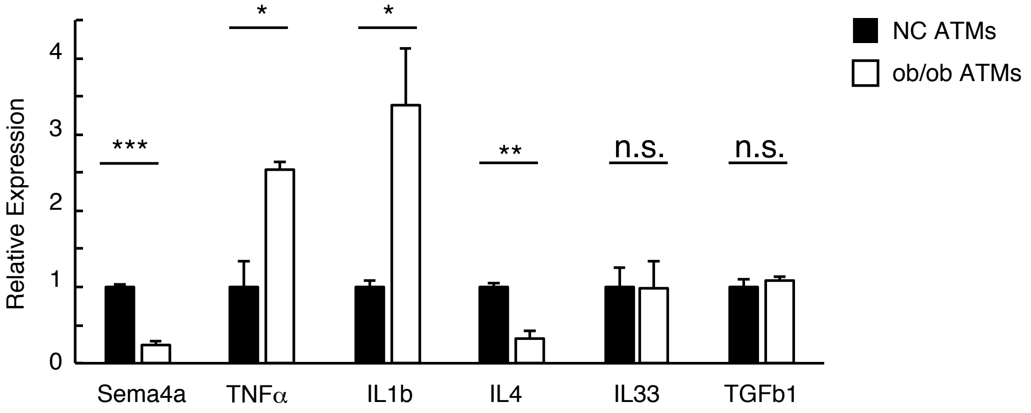


Supplementary figure 2. ob/ob ATMs have inflammatory phenotype compared with NC ATMs. Microarray analysis of the expression of genes encoding anti-inflammatory or inflammatory molecules and cytokines related to M2 phenotype by the adipose F4/80$^+$CD11b$^+$ macrophages from NC and ob/ob mice. Data are means ± SEM of three independent experiments. *$P<0.05$, **$P<0.01$ and ***$P<0.001$.

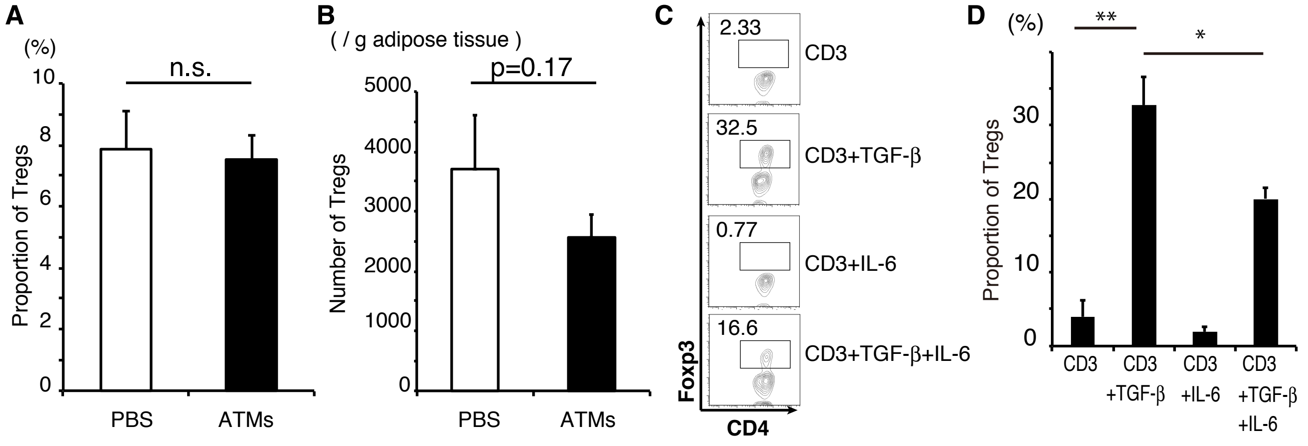


Supplementary figure 3. Administration of NC ATMs into obese adipose tissue have no effect on the differentiation and proliferation of adipose Tregs.

(A) Purified ATMs from NC mice were transferred into one side of epididymal adipose tissues in ob/ob mice as shown in figure 5A. Percentages of adipose Tregs among $CD3^{+}CD4^{+}$ T cells were analysed by FACS. (B) Numbers of Adipose Tregs were compared between ATM transferred side of adipose tissue and the opposite side of adipose tissue. Samples were measured in duplicate. Data are mean ± SEM of two independent experiments. (C) FACS analysis of the proportion of Tregs induced by ATMs in the presense of TGF-β and/or IL-6. (D) The proportion of Tregs calculated from results in (C). Data are mean ± SEM of two independent experiments.

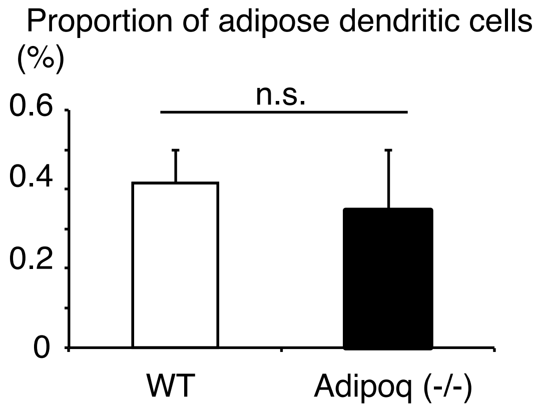


Supplementary figure 4. Effects of adiponectin deficiency on adipose tissue dendritic cells. Proportion of adipose tissue dendritic cells obtained from 10 to 14-week-old wild-type (WT) and adiponectin deficient mice [Adipo(-/-)]. Data are mean ± SEM (n=7 for WT, n=3 for Adipo(-/-)).

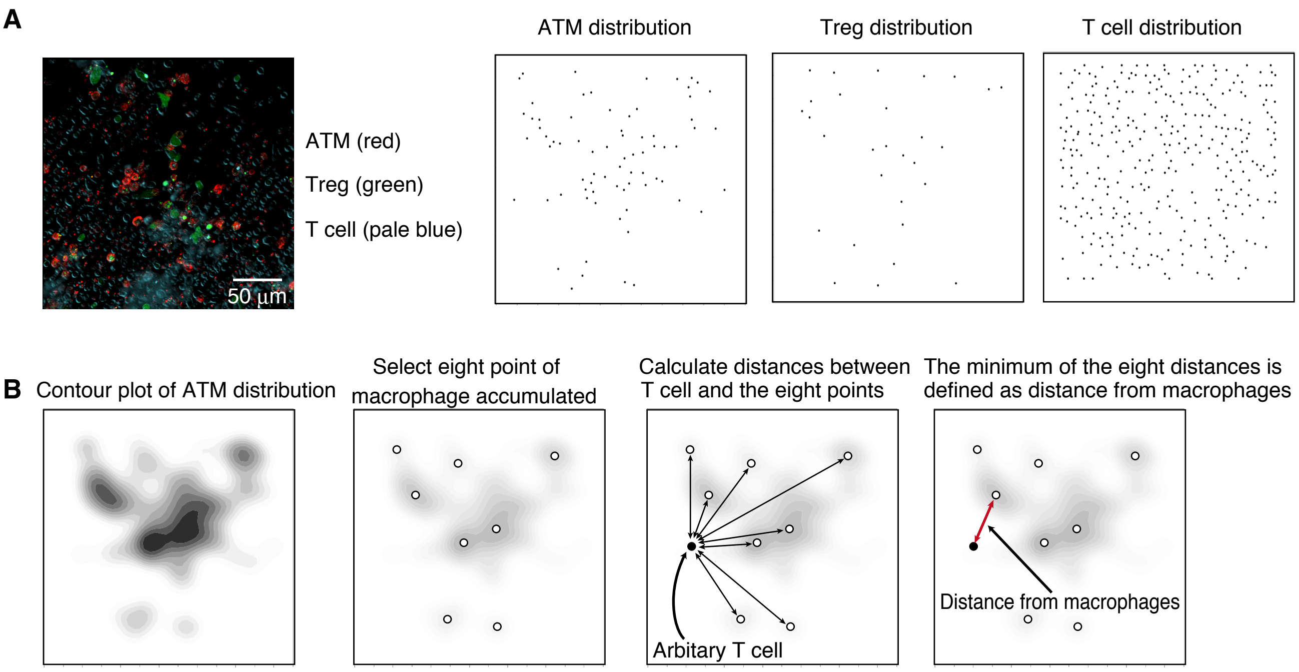


Supplementary figure 5. The method used to determine the distance between ATMs and T cells

(A) Representative image of co-cultured T cells (far left) and representative IMARIS spot data (left: ATM, right: Treg, far right: T cell). CMTPX labeled ATMs (red) and $CD4^{+}Foxp3^{-}$ T cells (pale blue) from Foxp3-EGFP mice were cultured, followed by confocal microscopy. The raw data were processed with IMARIS software and each cell was substituted for IMARIS spot automatically with manual correction.

(B) Contour plot of the distribution of each cell was obtained using the JMP software based on the position data of IMARIS spot (far left). Eight local maximum points were selected for substituting of numerous macrophages (left). The distance between the selected point and T cells was measured (right). The minimum value was defined as the distance from macrophages (far right).
